# First-principles investigations of As-doped tetragonal boron nitride nanosheets for toxic gas sensing applications

**DOI:** 10.1039/d4na00739e

**Published:** 2024-11-21

**Authors:** Kamal Hossain, Mohammad Tanvir Ahmed, Rabeya Akter Rabu, Farid Ahmed

**Affiliations:** a Department of Physics, Khulna University of Engineering & Technology Khulna 9203 Bangladesh; b Department of Physics, Jashore University of Science and Technology Jashore 7408 Bangladesh tanvir.phy43@gmail.com; c Department of Physics, Bangladesh Army University of Science and Technology Khulna 9204 Bangladesh; d Department of Physics, Jahangirnagar University Savar Dhaka 1342 Bangladesh

## Abstract

Pristine and arsenic-doped tetragonal boron nitride nanosheets (BNNS and As-BNNS) have been reported as potential candidates for toxic gas sensing applications. We have investigated the adsorption behavior of BNNS and As-BNNS for CO_2_, H_2_S, and SO_3_ gas molecules using first-principles density functional theory (DFT). Both BNNS and As-BNNS possess negative cohesive energies of −8.47 and −8.22 eV, respectively, which indicates that both sheets are energetically stable. Successful adsorption is inferred from the negative adsorption energy and structural deformation in the vicinity of the adsorbent and adsorbate. As-doping results in a significant increase in adsorption energies from −0.094, −0.175, and −0.462 eV to −2.748, −2.637, and 3.057 eV for CO_2_, H_2_S and SO_3_ gases, respectively. Due to gas adsorption, the electronic bandgap in As-BNNS varies by approximately 32% compared to a maximum of 24% in BNNS. A notable fluctuation in the energy gap and electrical conductivity is seen, with ambient temperature being the point of maximal sensitivity. For SO_3_, the maximum charge transfer during adsorption in BNNS and As-BNNS is determined to be 0.08|*e*| and 0.25|*e*|, respectively. Due to the interaction with gases, all structures exhibit an extremely high absorption coefficient on the order of 10^4^ cm^−1^ with minimal peak shifting. Additionally, doping an As atom on BNNS' surface remarkably improved its ability to sense CO_2_, H_2_S, and SO_3_ gasses.

## Abstract 2

The adsorption of CO_2_, SO_3_, and H_2_S gases on pristine and As-doped tetragonal boron nitride sheets (BNS) has been studied. As-doping significantly improves BNS's adsorption process.

## Graphical abstract

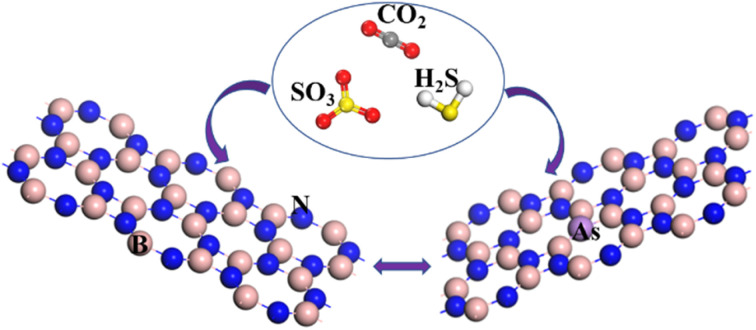

## 1. Introduction

In today's cities, rapid industrialization has led to substantial pollution from byproducts such as hazardous gasses and heavy metals, among others. Toxic gases and other vapor constituents can be identified with the help of gas-detecting technologies. The gas sensor device applications include pollution control, public and military safety, production, agriculture, tracking the environment, and healthcare diagnosis.^1–4^ Scientists report the presence of very poisonous gases, such as CH_4_, CO, SO_2_, CO_2_, NO, NH_3_, CH_3_OH, H_2_S, O_3_, PH_3_, and COCl_2_.^5–8^ Among these, H_2_S is a poisonous, explosive, and acidic gas that can seriously and irreversibly damage the nervous system.^9^ SO_3_ vapor is the most harmful to breathe in and can seriously burn the stomach, esophagus, and mouth.^10^ Furthermore, CO_2_ impacts human health due to its basic asphyxiant properties and is only slightly harmful when inhaled.^11^ The atmospheric pollutant CO_2_ is largely created by the ongoing expansion of vehicles and businesses, and it has a detrimental effect on our way of life and health.^12^ Different adsorbents have been employed to study the sensing mechanism of CO_2_, H_2_S, and SO_3_ gases.^13–16^

Gas sensing technology is therefore greatly impacted by the search for more affordable, ecologically safe, and effective sensors. Many materials, including conducting, semiconducting, and nanomaterials like graphene, have been proposed by research groups as possible gas sensors.^17–22^ Since the discovery of graphene, two-dimensional (2D) materials have drawn a lot of attention. However, because of its poor sensitivity, we must discover new substitute 2D materials for gas sensing.^23^ For gas sensing applications, graphene 2D sheets with various crystalline structures—such as hexagonal and tetragonal—are readily accessible and often utilized.^24–27^ Similar to graphene, boron nitride (BN) may exist in phases like the recently created tetragonal BN and hexagonal graphene-type BN (g-BN or h-BN).^28,29^ The h-BN nanosheets, sometimes called white graphene, show great promise due to their ability to sense gases.^30–33^ BN layers have generally been synthesized in the hexagonal phase and used as gas sensors because of their extraordinary structural and chemical properties, huge band gap, and amazing thermal stability.^32–34^ Numerous gas molecules, including NO_2_, NO, NH_3_, CO, CH_4_, H_2_, and others, have been studied for their adsorption on the surface of h-BN using both computational and experimental approaches.^35–40^ However, intrinsic 2D h-BN has limited reactivity for some inert gases.^41^

The most common method of improving the sensing performance on BN layers is element doping. Al and Ga-doped BN sheets have shown good halomethane sensitivity, whereas Al and Ga-doped BN nanotubes had substantial contact with NH_3_ gas.^42,43^ Higher adsorption energy has been observed in Co and Mn-doped BN layers when they interact more strongly with the gases CH_4_, H_2_S, NH_3_, O_3_, PH_3_, and SO_2_.^5^ For the adsorption of common SF_6_ decomposition gases, such as H_2_S, SO_2_, SOF_2_, and SO_2_F_2_, Sheng-Yuan Xia *et al.* suggested a Rh-doped h-BN (Rh-BN) monolayer as the perfect sensor.^44^ Moladoust *et al.* have created an Al and Si-doped BN nanostructure that exhibited greater adsorption energy for phosgene gas than a pure BN structure.^45^ An As-doped BN monolayer for SO_2_F_2_ gas molecules demonstrated good adsorption specificity, high sensitivity, and a short recovery time, as demonstrated by Yunfeng Long *et al.*^46^ Tetragonal BN nanosheets (BNNS) are one of the boron nitride allotropes whose physical and adsorption properties have been studied the least. Sakib *et al.* used DFT to study the adsorption properties of BNNS in the tetragonal phase and discovered negative adsorption energy for thioguanine, an anticancer drug.^47^ Tetragonal BNNS has a large semiconducting bandgap, which has been observed, and it has been shown that electrical conductivity changes by 8% under compressive stress.^48^

Significant research attention has been drawn to the novel applications of an emerging class of 2D materials for ecologically hazardous gas sensing applications. We investigated As-doped tetragonal boron nitride nanosheets as a new kind of adsorbent for use in gas sensing. Due to the scarcity of studies on how the adsorption properties are affected by As atom doping in pure BNNS, we have selected this dopant. Using the DFT approach, we have computed the structural, adsorption, and optoelectronic characteristics of As-doped BNNS and pristine BNNS for CO_2_, H_2_S, and SO_3_ gas adsorption.

## 2. Computational framework

Using DFT and the Cambridge Serial Total Energy Package (CASTEP) algorithm, first-principles calculations of the geometrical, optical, and electronic response of pristine BNNS and As-BNNS during the adsorption of CO_2_, H_2_S, and SO_3_ gas molecules were performed in this study.^49^ Using the Broyden–Fletcher–Goldfarb–Shanno (BFGS) minimization technique, all atoms can be relaxed during the optimization process.^50^ The wave function of valence electrons is simulated by the CASTEP algorithm using a combination of plane wave basis sets with kinetic energy less than the cut-off energy.^51^ The exchange–correlation potential is considered in the Perdew–Burke–Ernzerhof (PBE) functional of the Generalized Gradient Approximation (GGA-PBE)^52^ functional.

Optimizing the pristine BNNS structure, the impact of many plane-wave cutoff energies—350 eV, 400 eV, 450 eV, and 500 eV—was investigated; the lowest energy was reached at 500 eV. Subsequently, ultra-soft pseudo-potentials^52^ with a kinetic energy cutoff of 500 eV were employed throughout the simulation. Here, we have performed Brillouin zone integration for structural optimization of unit cells using a 6 × 6 × 1 mesh Monkhorst–Pack grid.^53^ To have enough space for adsorption of gas molecules, we built a 3 × 2 × 1 supercell with a 20 Å vacuum space along the BNNS structure's *z*-direction. This allowed us to sample the Brillouin zone using a 2 × 3 × 1 *k*-point Monkhorst–Pack mesh. In the whole study, Grimme's dispersion correction scheme has been employed to take the long-range van der Waals interaction into account.^54^ For all geometric optimizations, the convergence tolerance conditions are set at fine quality. The self-consistent estimation relies on the self-consistent convergence of the total energy of 1 × 10^−5^ eV per atom, the maximum force on the atom of 0.03 eV Å^−1^, the maximum ionic displacement within 0.001 Å, and the maximum stress within 0.05 GPa.

Electronic band structure, density of states, electronic density difference, Mulliken charge population, and optical characteristics were all carefully examined to thoroughly characterize optoelectronic responses. The B-2s^2^ 2p^1^, N-2s^2^ 2p^3^, H-1s^1^, S-3s^2^ 3p^4^, As-4s^2^ 4p^3^, C-2s^2^ 2p^2^, and O-2s^2^ 2p^4^ valence electron configurations were employed for pseudo atomic calculations. To compute optical properties, the complex dielectric constant was evaluated using the Kramer–Kronig relationship.^55^ Since it provides access to new optical parameters, the complex dielectric function is a crucial component of optical properties. To study the sensitivity of the nanosheets toward gas molecules, the adsorption energy (*E*_ads_) was calculated using the following equation^56^

1 *E*_ads_ = *E*_BNNS+gas_ − *E*_BNNS_ − *E*_gas_

where the energy of the gas-adsorbed nanosheet, the nanosheet (without the gas molecule), and the gas molecule is represented, respectively, by the symbols *E*_BNNS+gas_, *E*_BNNS_, and *E*_gas_. The negative adsorption energy^33^ confirms the stability of the adsorbed gas on the nanosheets. Nonetheless, the adsorption locator module was utilized to achieve the steady adsorption configuration.^39–41^

Pristine BNNS and As-BNNS stability was examined by calculating their binding energy or cohesive energy using the following equation^57^2
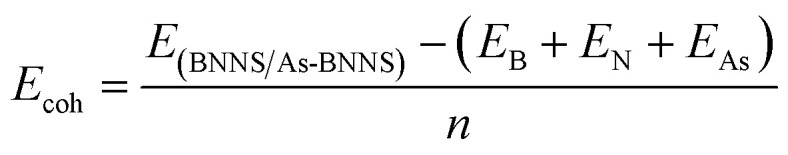
where *E*_BNNS/As-BNNS_, *E*_B_, *E*_N_, *E*_As_, and *n* are the energy of BNNS or As-BNNNS, isolated boron, isolated nitrogen, isolated arsenic atom, and the total number of atoms respectively.

## 3. Results and discussion

### 3.1 Geometry analysis

**Fig. 1:**
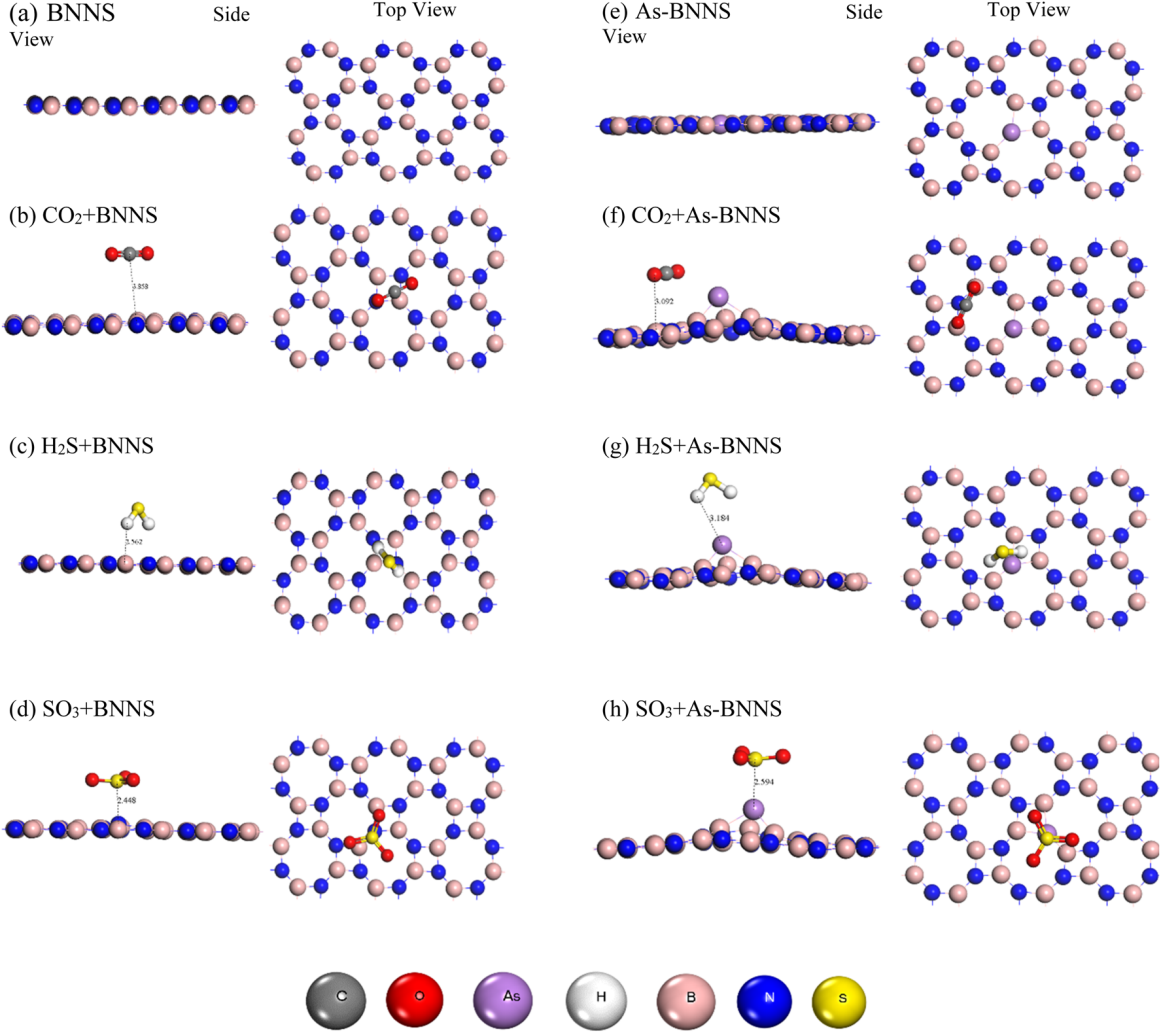
Optimized structures (side and top views) of pristine BNNS, As-BNNS, and gas adsorbed BNNS and As-BNNS.

The optimized BNNS, As-BNNS, and gas adsorbed-BNNNS structures are shown in Fig. 1 in both side and top views. Table 1 lists the average bond lengths for gas molecules, As-BNNS, and pure BNNS. The B–N bond length in pristine BNNS is stated to be 1.449 Å, which is similar to the earlier finding.^47^ With 48 atoms, the 2D pure BNNS forms 8 hexagons across the layers. We observed modifications in the bond length due to arsenic doping instead of nitrogen. The lengths of the As–B bonds are 1.867 Å and 1.794 Å. This might result from the interaction of the arsenic atom with its varied size and charge distribution.^46^ The bond lengths in CO_2_, H_2_S, and SO_3_ optimized gas molecules are 1.181 Å, 1.351 Å, and 1.438 Å for C=O, H–S, and S=O respectively. These findings are consistent with the earlier research.^12,58–62^ We observed a slight alteration in the bond length of both the gas molecules and BNNS following the gas adsorption. The length of the B–N bond varies from 1.79% to 4.07%, which might be a key sign of how interactive BNNS is with these gas molecules. In CO_2_ + BNNS, H_2_S + BNNS, and SO_3_ + BNNS, the B–N bond lengths are 1.475 Å, 1.477 Å, and 1.508 Å, in that order. The measured modifications in gas molecules are 1.352 Å and 1.442 Å for the H–S and S=O bonds, respectively. The change in the average bond length of the adsorbent and adsorbate signifies the interaction between them.^62,63^ Compared to H_2_S and CO_2_, a greater interaction with SO_3_ is shown by the greater alteration in the B–N bonds caused by SO_3_ adsorption. The gas adsorption in the instance of As-BNNS modifies the B–N bond length from 2.01% to 2.12%. This demonstrates that As-BNNS is interactive with gas molecules. However, because of the 8.78% to 9.75% increase in As–B bond length, the interaction is higher in the presence of an arsenic atom when compared to BNNS. We measured the C=O, H–S, and S=O bond lengths in gas-adsorbed As-BNNS to be 1.181 Å, 1.351 Å, and 1.446 Å, respectively.

**Table 1:** Bond length (Å) and cohesive energy (*E*_coh_) of the optimized structures

Molecules	Bond lengths					*E*_coh_ (eV)
	B–N	As–B	C=O	H–S	S=O	
BNNS	1.449	—	—	—	—	−8.47
As-BNNS	1.508	1.867, 1.794	—	—	—	−8.22
CO_2_	—	—	1.181	—	—	—
H_2_S	—	—	—	1.351	—	—
SO_3_	—	—	—	—	1.438	—

This suggests that a greater variation in the S=O bond length causes As-BNNS to interact more with SO_3_. One of the primary issues was the geometrical stability following the arsenic doping in the pure BNNS layer. Using eqn (2), we have determined the cohesive energy of the adsorbents to elucidate the stability. The energetic stability of BNNS and As-BNNS is shown by the negative values of *E*_coh_, which were determined to be −8.47 and −8.22 eV, respectively. Hence, the tetragonal BNNS remains stable after As-doping with a slight distortion in the geometrical structure.^46^

A traditional method for investigating the adsorption performance and longer lifespan of any sensor device is the deformation study of the adsorbent layer. As a result, we use the following equation (eqn (3)) to determine the deformation of the BNNS and As-BNNS layers caused by gas adsorption by measuring the energy of the adsorbents that are deformed and undeformed.3
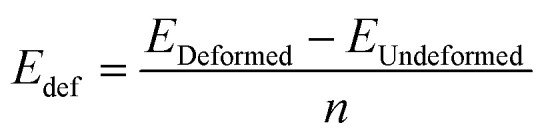
where *E*_Undeformed_ is the energy of the adsorbent layer and *E*_Deformed_ is the energy of the deformed adsorbent after gas adsorption. After gas adsorption we removed the gas molecules and the energy of the deformed adsorbent has been calculated. A sensor device with a longer lifespan is more suitable the less the adsorbent deforms when there is an adsorbate present. When CO_2_ is adsorbed, the pristine BNNS shows the least amount of deformation. When H_2_S and SO_3_ are adsorbed, the deformation tends to increase and is greatest for SO_3_. This suggests that, in comparison to alternative options, using BNNS as a CO_2_ gas sensor is adequate. The SO_3_ adsorption in the As-BNNS instance demonstrates the least amount of base layer deformation, with a deformation energy of 0.051 eV. The As-BNNS experiences a comparable deformation with a deformation energy of 0.053 eV when CO_2_ and H_2_S are adsorbed. This suggests that As-BNNS is a suitable and longer-lasting SO_3_ gas sensor. It is observed that in the presence of gas molecules, the deformation of BNNS is smaller and varies more than that of As-BNNS.

### 3.2 Adsorption of CO_2_, H_2_S and SO_3_ gases

When it has a negative value, adsorption energy describes the direct interaction of base layers with gas molecules.^64^ All of the gas molecules are physically adsorbed without creating covalent bonds with the adsorbent, as shown in Fig. 1 and Table 2. We have obtained the adsorption energy and adsorption length as shown in Table 3. All of the gas molecules show negative adsorption energy in BNNS, suggesting that they have successfully adsorbed on the material.

**Table 2:** Bond length (Å) in optimized gas adsorbed BNNS and As-BNNS, and deformation energy (*E*_def_) per atom

Elements	Bond length					*E*_def_ (eV per atom)
	B–N	As–B	C=O	S–H	S=O	
CO_2_ + BNNS	1.475	—	1.181	—	—	2.6 × 10^−6^
H_2_S + BNNS	1.477	—	—	1.352	—	9.75 × 10^−6^
SO_3_ + BNNS	1.508	—	—	—	1.442	3.08 × 10^−3^
CO_2_ + As-BNNS	1.476	2.049, 1.990	1.181	—	—	0.053
H_2_S + As-BNNS	1.476	2.042, 1.984	—	1.351	—	0.053
SO_3_ + As-BNNS	1.477	2.031, 1.971	—	—	1.446	0.051

**Table 3:** Adsorption energy (*E*_ad_) and adsorption length of the BNNS and As-BNNS sheets for gas molecules

Adsorbed gas	*E*_ad_ (eV)		Adsorption length (Å)	
	BNNS	As-BNNS	BNNS	As-BNNS
CO_2_	−0.094	−2.748	3.858	3.092
H_2_S	−0.175	−2.637	2.562	3.184
SO_3_	−0.462	−3.057	2.448	2.594

Following adsorption, the BNNS planar structure is retained with less deformation. These findings are comparable to research on deformation energy. Comparatively, SO_3_ with a maximum adsorption energy of −0.462 eV shows that SO_3_ gas is adsorbed more strongly than the others. The adsorption energy finding is followed by the minimum adsorption length for SO_3_. For CO_2_, H_2_S, and SO_3_ gas molecules, however, the comparable adsorption energy and adsorption length indicated that BNNS is an effective adsorbent.

The BNNS layer's planar structure is somewhat distorted when one arsenic atom is doped. Furthermore, the adsorption of gas molecules enhances this deformation, brought about by Table 2's higher deformation energy. Table 3 demonstrates how arsenic doping improved the adsorption characteristics in As-BNNS relative to BNNS, with greater adsorption energy. As-BNNS, like BNNS, shows maximum adsorption for SO_3_ gas at a minimum adsorption length of 2.594 Å and a higher negative value of *E*_ad_ of −3.057 eV. Overall, the BNNS and As-BNNS are suitable adsorbents for CO_2_, H_2_S, and SO_3_ gas molecules.

We have investigated the recovery time by following the equation for BNNS and As-BNNS after the CO_2_, H_2_S, and SO_3_ gas adsorption.^65^4
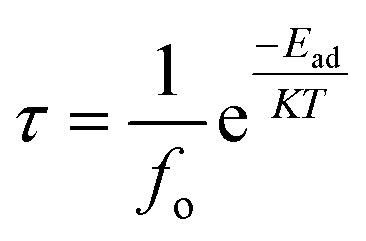
where *K*, *T*, and *f*_o_ stand for Boltzmann's constant, temperature and UV radiation frequency (10^12^ to 3 × 10^14^ Hz), respectively. One of the most important factors in enhancing the effectiveness of gas-sensing devices is quick recovery. After the gas adsorption, the adsorbent is typically recovered by chemical exposure and irradiation with high-intensity electromagnetic radiation. It is possible to recover the weak or intermediate-level interaction between gas molecules using UV light. Chemical reactions involving reagents including HCl, NaOH, HNO_3_, and NaHCO_3_ work better for heavy adsorption in recovering the adsorbent for use in subsequent processes.^66–69^Table 4 shows that while the As-BNNS layer has a longer recovery time than the BNNS layer, the greater adsorption results in a longer recovery time. Sensing CO_2_, H_2_S, and SO_3_ gases will be simpler with BNNS as a sensor than As-BNNS. However, the chemical reaction can be employed in the event of hard adsorption and extended recovery time for As-BNNS.

**Table 4:** Recovery time *τ* (s) for different adsorbates in the BNNS and As-BNNS

Adsorbed gas	BNNS		As-BNNS	
	*f*_o_ = 10^12^	*f*_o_ = 3 × 10^14^	*f*_o_ = 10^12^	*f*_o_ = 3 × 10^14^
CO_2_	3.94 × 10^−11^	1.31 × 10^−25^	3.03 × 10^34^	1.01 × 10^20^
H_2_S	9.03 × 10^−10^	3.01 × 10^−24^	3.92 × 10^32^	1.30 × 10^18^
SO_3_	6.40 × 10^−5^	2.13 × 10^−19^	5.02 × 10^39^	1.67 × 10^25^

### 3.3 Electronic properties

**Fig. 2:**
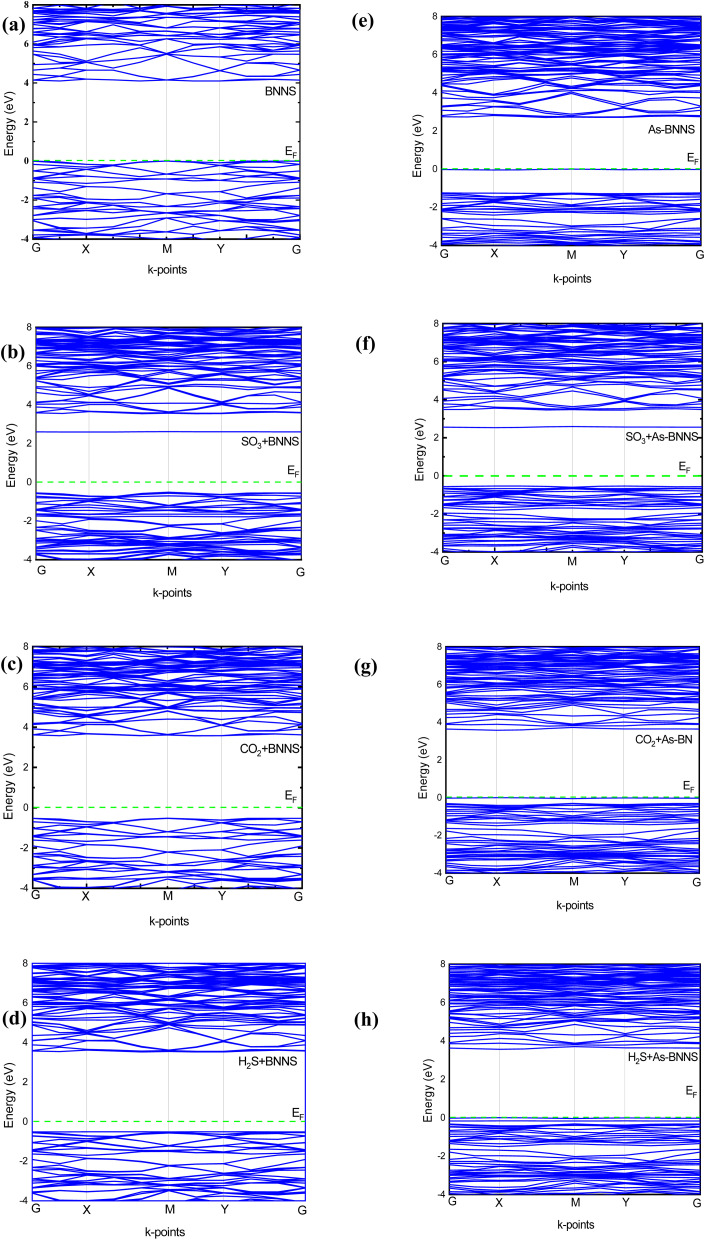
Electronic band structures of BNNS, As-BNNS, gas adsorbed BNNS and As-BNNS structures.

The produced electronic band structure, which is based on the accumulated adsorbate molecules in the adsorbed system, describes the clear variations in the energy gap between the valence band and conduction band. To gain a comprehensive understanding of their electrical response, we carried out electronic band structure (EBS) analysis for the pristine and As-doped BNNS structures as well as the gas-adsorbed pristine and As-doped BNNS structures which shed light on their optical properties. To examine the exchange–correlation potential for electronic structure analysis using CASTEP, we concentrated on GGA-PBE schemes. A range of physicochemical factors, such as the kind of conductor, the electrochemical potential of various species, and the activity of surface reactions, may be explained by the EBS concerning the Fermi level.^70^Fig. 2 provides a detailed depiction of the adsorbent and adsorption system's EBS series of energetic lines along high symmetry points as *G*–*X*–*M*–*Y*–*G*. Due to their symmetry with spin polarization, all of the band structures are only depicted for up-spin circumstances.

Pure BNNS and gas-adsorbed BNNS structures have an indirect band gap, with the valence band maximum (VBM) at point *M* in *K*-space and the conduction band minimum (CBM) between the *G* and *X* points. The calculated bandgaps along with Fermi energy for the investigated structures are tabulated in Table 5. The prohibited passage of electrons from the valence band to the conduction band is described by the enormous bandgap of 4.098 eV seen in pristine BNNS, which is comparatively smaller than that of the h-BN monolayer,^71^ making the tetragonal phase more suitable for numerous optoelectronic studies. The CO_2_, H_2_S, and SO_3_ adsorbed pristine BNNS had electronic bandgaps of 4.088 eV, 4.035 eV, and 3.114 eV, respectively. We have reported the minimum and maximum variation of bandgaps between pristine and gas-adsorbed BNNS is found to be 0.2% and 24% respectively. This might indicate that gas molecules have been adsorbed, causing an insulator-to-semiconductor transition. The dispersion of the energy band and the change in bandgap energy during gas adsorption demonstrate the high interaction of pure BNNS to gas molecules.

**Table 5:** Calculated bandgap (*E*_g_), Fermi energy (*E*_F_), work function (*Φ*), electrical conductivity (*σ*) and sensitivity of the adsorbents and their gas-adsorbed complexes

Elements	*E*_g_ (eV)	*E*_F_ (eV)	*Φ* (eV)	Δ*Φ* (eV)	*σ* (*A* × ohm^−1^ m^−1^)		
					300 K	400 K	500 K
BNNS	4.098	−1.131	1.1313	—	0.924	0.942	0.954
CO_2_ + BNNS	4.088	−4.129	4.1288	2.997	0.924	0.942	0.954
H_2_S + BNNS	4.035	−4.230	4.2302	3.099	0.925	0.943	0.954
SO_3_ + BNNS	3.114	−2.163	2.1628	1.032	0.942	0.956	0.965
As-BNNS	2.708	−1.375	1.3752	—	0.949	0.961	0.969
CO_2_ + As-BNNS	3.576	−1.350	1.3501	0.025	0.933	0.949	0.959
H_2_S + As-BNNS	3.567	−1.496	1.4962	0.121	0.933	0.950	0.959
SO_3_ + As-BNNS	3.066	−2.523	2.5227	1.148	0.942	0.956	0.965

In the case of As-BNNS, we observed an indirect semiconducting bandgap of 2.708 eV with the VBM between *G* and *X* points and CBM at *M* points. However, all the gas-adsorbed As-BNNS exhibit a direct band gap nature with the CBM and VBM at *M* point. The bandgaps for CO_2_, H_2_S, and SO_3_ adsorbed As-BNNS are found to be 3.576 eV, 3.567 eV, and 3.066 eV respectively. In this case, the minimum and maximum variations are 13% and 32% respectively. The semiconducting bandgap of As-BNNS increases with the adsorption of gas molecules. This indicates the opposite interaction of gas molecules with As-BNNS compared to the pure BNNS layer. This may result from the increase of bandgap in the presence of gas molecules.

**Fig. 3:**
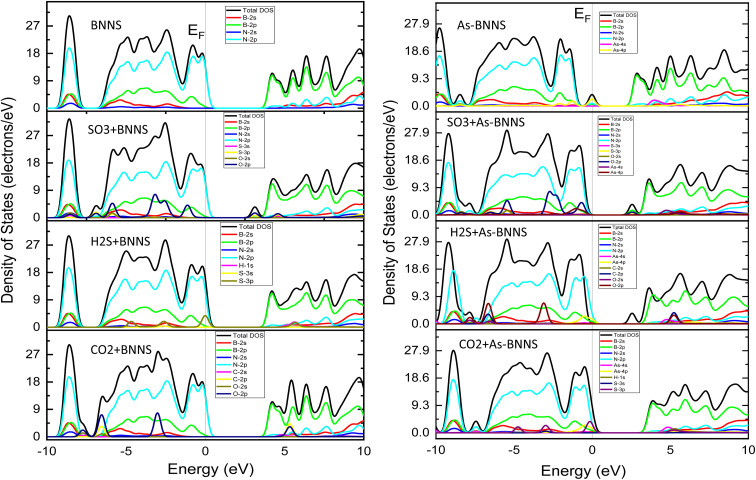
The density of states (DOS) and projected density of states (PDOS) of BNNS, As-BNNS, gas-adsorbed BNNS and As-BNNS structures.

Overall, the change in the EBS suggested that the pristine BNNS and As-BNNS have strong interaction with the CO_2_, H_2_S, and SO_3_ gas molecules. However, this interaction is stronger in the case of As-BNNS resulting from the large variation in bandgaps than BNNS. In addition to EBS, a material's DOS and PDOS are employed to determine its electronic nature. The final clue as to whether a material is an insulator, conductor, or semiconductor is the presence of electron states close to the Fermi level *E*_F_. The contribution and interactions of the orbital electrons determine the type and creation of EBS. With the significance of DOS and PDOS in mind, we computed the total DOS and PDOS for gas-adsorbed BNNS and As-BNNS structures as well as BNNS and As-BNNS structures throughout the energy range of −10 eV to 10 eV, as shown in Fig. 3.

We observed that DOS is absent in BNNS throughout a wide energy range indicating BNNS's broad bandgap compared to others. The bandgap was decreased and the valence and conduction bands moved in the direction of the Fermi level as a result of the gas adsorption in BNNS. We have found that in BNNS, B-2p and N-2p orbital electrons predominate in the valence band and conduction band, following the PDOS. Along with B-2p and N-2p, the O-2p orbital electrons in CO_2_-BNNS contribute significantly to the valence and conduction bands, compared to C-2p. The addition of S-3p orbital electrons mostly drove the energy bands toward the Fermi level in the H_2_S-BNNS instance, reducing the bandgap simultaneously. More than half-occupied O-2p and S-3p orbital electrons have been shown to contribute significantly to the greatest shift of the valence band and conduction band observed in SO_3_-BNNS.

Doping allowed us to see the semiconducting bandgap and electron states that were very close to the Fermi level. However, energy bands in gas-adsorbed As-BNNS moved out from the Fermi level, creating a larger bandgap. The As-4p orbital electrons predominate in the VBM and CBM, but all of the gas molecules in As-BNNS and their valence orbitals follow almost identical laws to those in BNNS. BNNS and As-BNNS, however, exhibit high interaction for the studied gas molecules, according to the electronic response revealed by the study of DOS and PDOS. This interaction is increased by doping the As atom in pure BNNS.

The variation in the bandgap energy of any material influences the electrical conductivity. In semiconducting materials, the electrical conductivity reduces with increasing electronic bandgap. Semiconducting materials have a positive temperature coefficient of conductivity. Such variation of electrical conductivity with bandgap and temperature can be observed in the following equation:^72^5
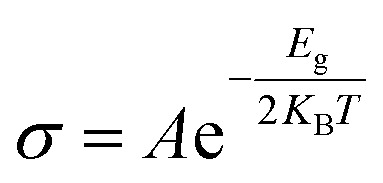
where *A* is a constant term which has not been determined yet since the structures are not synthesized experimentally. Hence only the exponential term was calculated to show the variation of electrical conductivity with respect to *E*_g_. Therefore, the unit of *σ* has been written as *A* × ohm^−1^ m^−1^. Table 5 lists all of the computed electrical conductivity values for the compositions under investigation. We have observed that as bandgap energy decreases, electrical conductivity rises exponentially. When gas adsorption occurs on BNNS, the minimum bandgap and maximum conductivity are displayed by SO_3_ + BNNS. The adsorbent layer As-BNNS exhibits the lowest bandgap and highest conductivity during gas adsorption, and conductivity also decreases after gas adsorption. Overall, BNNS's electrical conductivity rises with gas adsorption, but As-BNNS exhibits the opposite behavior. Table 5 shows the opposite trend to the bandgap fluctuation based on the temperature change of electrical conductivity. Electrical conductivity rises as we go from 300 K to 500 K temperature. The As-BNNS structure's highest conductivity is discovered at 500 K. By displaying changes in electrical conductivity and resistivity when gas is present near the adsorbent, the gas sensor can identify the presence of gas molecules.

Any adsorbent's fluctuation in electrical conductivity is determined by the work function *Φ*, which gives a probability that charge carriers will be transported from the adsorbent surface. As a result, we noticed that the work function of adsorbent materials varied depending on whether gas molecules were present or not as follows:^73^

6 *Φ* = |*V*_∞_ − *E*_F_|

where *V*_∞_ refers to the potential at an infinite distance from the adsorbent surface which is considered as zero. Table 5 presents the work function-derived values in an ordered manner.

In comparison to the As-BNNS structure, the BNNS structure has a maximum charge escape probability and a minimum *Φ*. Following gas adsorption, As-BNNS is responsible for CO_2_ adsorption while BNNS has a minimal work function during SO_3_ adsorption. The gas adsorption resulted in a significant change in *Φ* which can be utilized to build work function-based sensors, where *Φ* of the complexes can be measured experimentally *via* the Kelvin method based on these variations in work function.^60^ The sensor can be calibrated with reference values of *Φ*, which can not only sense the presence of selected gases but also identify the type of toxic gas. The mathematical relation between the variation of conductivity and *Φ* is the Richardson–Dushman relation^74^ as follows:7
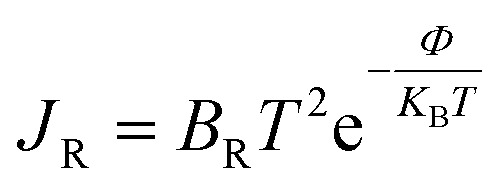
where *B*_R_ and *J*_R_ stand for the Richardson constant and the density of released charge carriers, respectively. According to the aforementioned equation, the number of escaping charge carriers rises exponentially as *Φ* decreases. Based on Table 5's observation of *Φ* fluctuation, the BNNS layers have a very high surface conductivity.

**Fig. 4:**
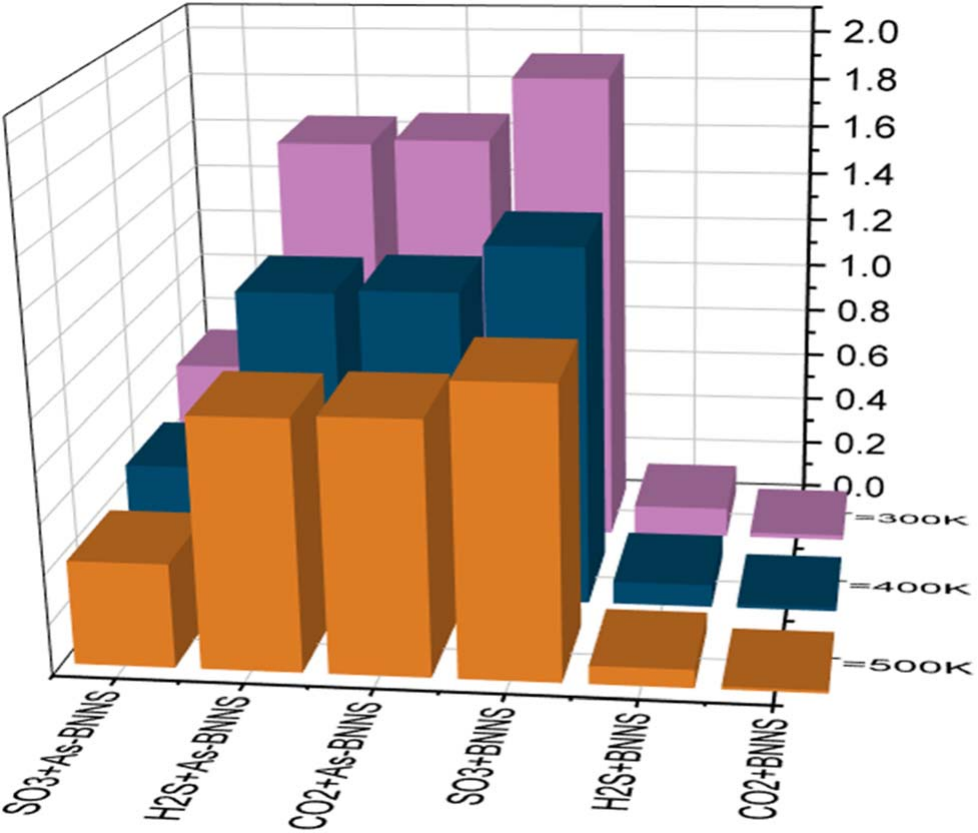
Sensitivity of gas adsorbed BNNS and As-BNNS structures at different temperatures.

The sensitivity of any adsorbent for certain gas molecules can be observed by detecting the variation of electrical conductivity in the presence and absence of gas molecules, which can be formulated as:^75^8
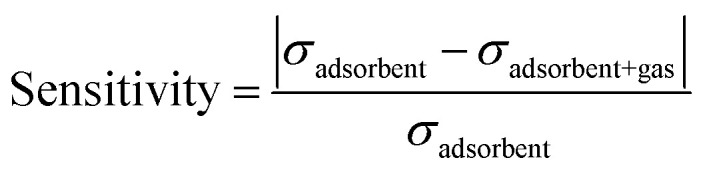
where *σ*_adsorbent_ and *σ*_adsorbent+gas_ are the conductivity of the adsorbent layers and adsorbent with gas respectively. The variation of sensitivity with the variation of gas molecules and temperature is illustrated in Fig. 4. The As-BNNS has the highest sensitivity to SO_3_ at 300 K out of all the gas adsorption structures. Furthermore, when the temperature rises from 300 K to 500 K, the sensitivity falls. Consequently, the As-BNNS may be effectively employed as a sensor medium for the adsorption of SO_3_ gas under ambient conditions.

**Fig. 5:**
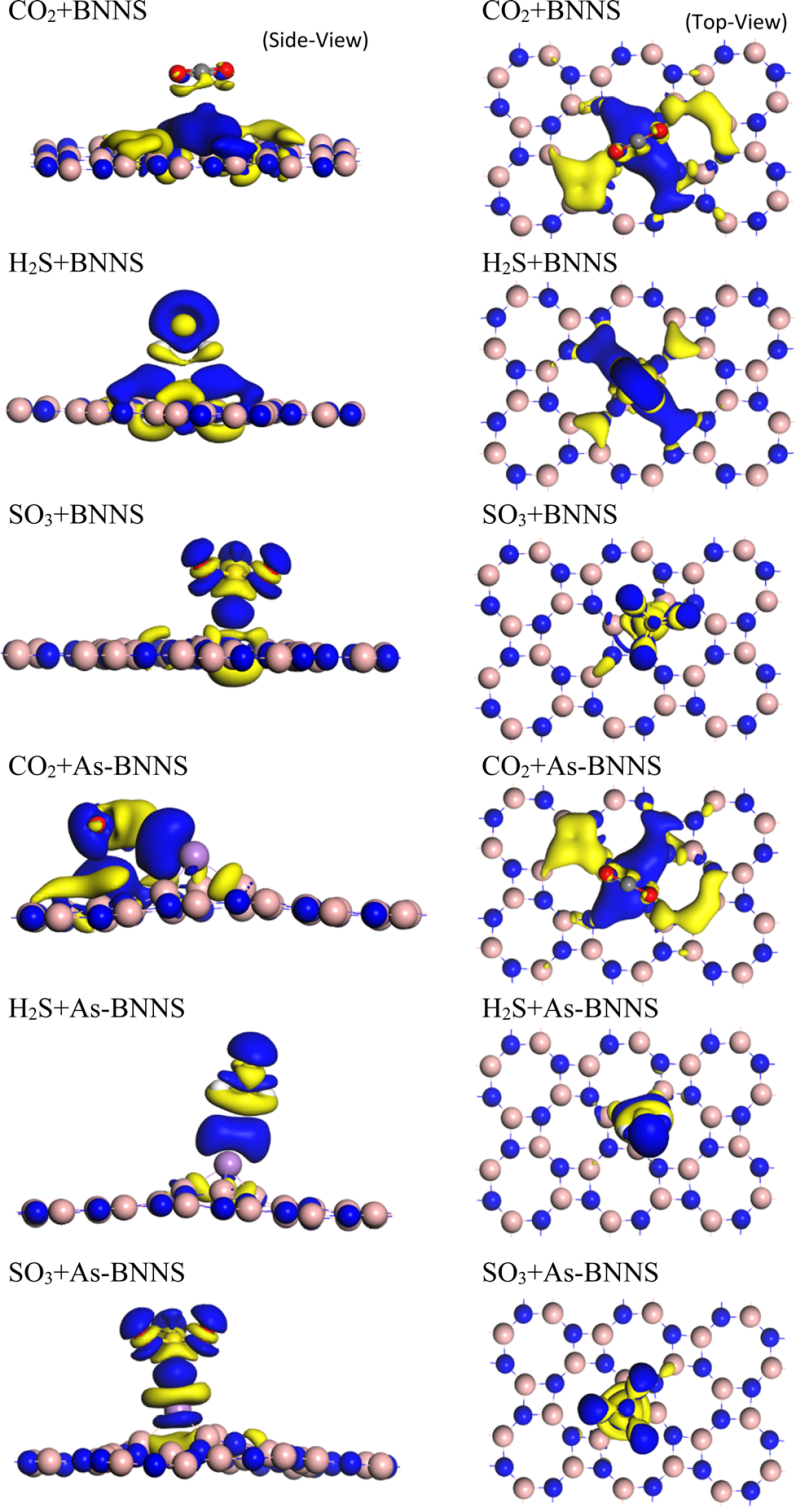
EDD mapping of gas adsorbed BNNS and As-BNNS structures at different temperatures.

We conducted Mulliken bond population analysis (MPA),^76,77^ a widely used method for learning more about a molecule's bonding activity. The calculated Mulliken charges in BNNS and gas-adsorbed BNNS are given in Table 6. As seen in Fig. 5, electron density difference (EDD) mapping provides a clear image of the fluctuation in electron density caused by gas adsorption. The yellow zone denotes an electron shortage, while the blue region indicates an electron buildup. The partially ionic nature of the B–N bonds in the pristine BNNS is indicated by charge analysis using MPA, which shows that an average of 0.87|*e*| charge is transferred from the B atoms to their neighboring N atoms within the sheet due to the N atom's higher electronegativity.

**Table 6:** Average Mulliken charge distribution of pristine BNNS and gas-adsorbed BNNS

Elements	BNNS	BNNS + CO_2_	BNNS + H_2_S	BNNS + SO_3_
B	0.87	0.869	0.867	0.873
N	−0.87	−0.87	−0.868	−0.868
H	—	—	0.17	—
C	—	0.97	—	—
O	—	−0.49	—	−0.80
P	—	—	—	—
S	—	—	−0.36	2.32

Following Mulliken charges in Table 7, there is only a little shift in the charges of B and N. H and C exhibit their characteristic electropositive nature in the gas adsorption structures, whereas O exhibits an electronegative nature. On the other hand, S has an electropositive character when O is present and an electronegative character when H is present.

**Table 7:** Mulliken charge distribution of As-BNNS and gas adsorbed BNNS

Elements	As-BNNS	As-BNNS + CO_2_	As-BNNS + H_2_S	As-BNNS + SO_3_
B	0.808	0.820	0.820	0.828
N	−0.854	−0.861	−0.861	−0.861
As	0.27	0.12	0.16	0.19
H	—	—	0.16	
C	—	0.94	—	—
O	—	−0.48	—	−0.83
P	—	—	—	—
S	—	—	−0.35	2.24

This may result from the S-3p^4^ orbital's behavior to receive or leave electronic charge to make a stable configuration. The comparison shows that SO_3_ > H_2_S > CO_2_ is the overall charge transfer from gas molecules to the adsorbent based on the total Mulliken charge of both the gas and the adsorbent. The EDD mapping result from the dispersion of charge density towards gas molecules follows this outcome. Though the quantity of charge transfer in As-BNNS is more than that of BNNS, charge transfer between the gas molecules and adsorbent still occurs in the same sequence.

**Fig. 6:**
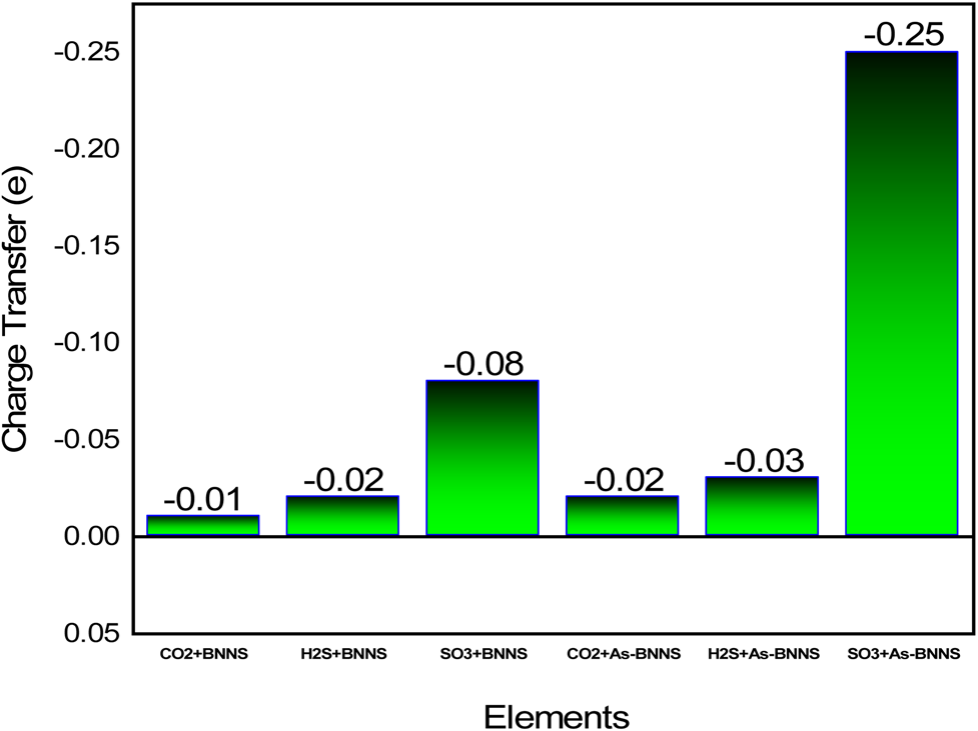
Charge transfer between the host layers and gas molecules.

Given the greater dispersion of the isosurfaces toward gas molecules, the EDD mapping is consistent with these observations. It is noted that the As-BNNS and gas-adsorbed As-BNNS structures exhibit comparatively greater changes in the partial charges of B and N. As, an electropositive element, exhibits partial positive charges in the structures of As-BNNS and gas-adsorbed As-BNNS. Following gas adsorption, the As atom primarily loses its charge to the BN sheet and the electronegative components of gas molecules. This being said, there is consistency between the EDD analysis and Mulliken charge. The main determinant of material interaction is charge transportation. This idea can help explain how gas molecules interact with the adsorbent layers. Fig. 6 illustrates the charge transfer between the adsorbent and gases. It is evident that during gas adsorption, the adsorbent layer leaves charge carriers behind, which act as donors and gas molecules as receivers. A maximum charge transfer of −0.08*e* is seen in the SO_3_ + BNNS structure. The As-BNNS structure has demonstrated similar outcomes in the charge transfer pattern. It can be shown from Fig. 6 that the doping of the As atom enhances the charge transfer from the adsorbent to gas. Overall, the interaction between the adsorbent layer and the gas molecules was improved by As atom doping.

### 3.4 Optical properties

#### 3.4.1. Dielectric constants

**Fig. 7:**
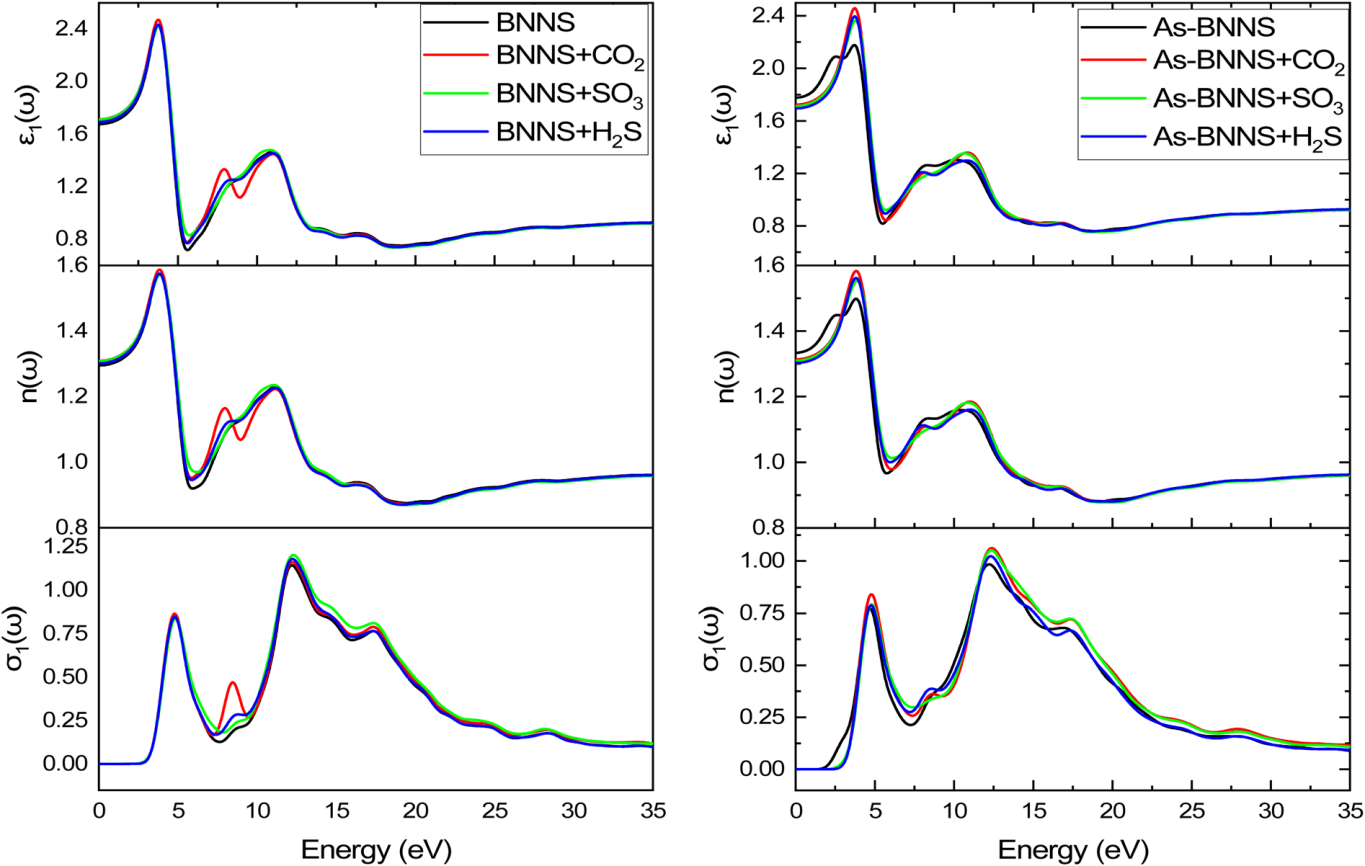
The real part of dielectric constant *ε*_1_(*ω*), refractive index *n*(*ω*), and conductivity *σ*_1_(*ω*) from top to bottom.

**Fig. 8:**
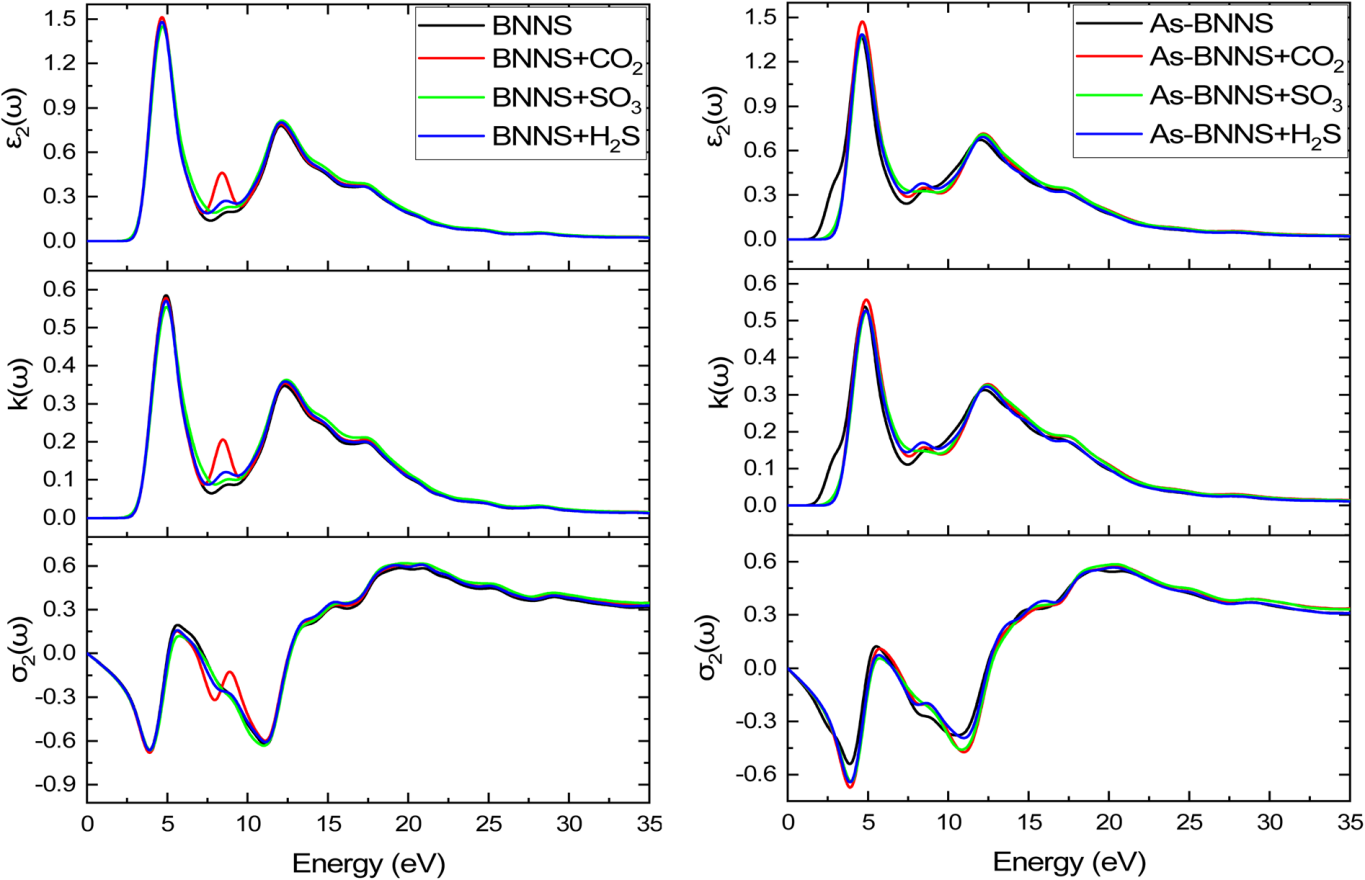
The imaginary part of dielectric constant *ε*_2_(*ω*), refractive index *k*(*ω*), and conductivity *σ*_2_(*ω*) from top to bottom.

**Fig. 9:**
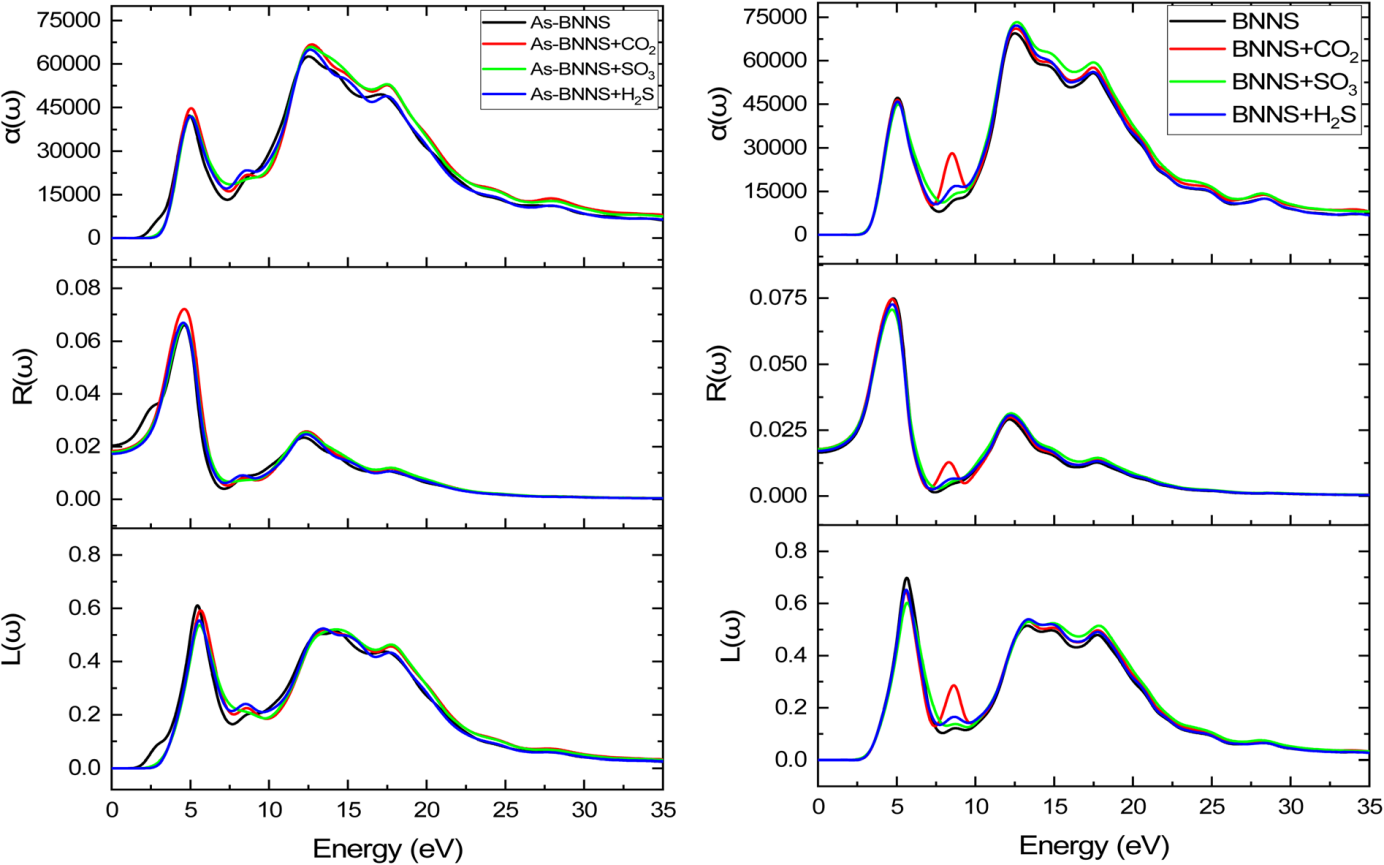
The calculated absorption coefficient *α*(*ω*), reflectivity *R*(*ω*), and loss function *L*(*ω*).

In gas sensing research, the study of the pure layer's and gas-adsorbed layer's optical properties aids in determining a layer's sensitive features. DFT is a valuable theoretical technique applied to many-body systems using the principles of quantum mechanics. All the frequency-dependent optical parameters of pure and gas adsorbed layers are depicted in Fig. 7–9. The optical characteristics mentioned above are mainly ascribed to the computation of the complex dielectric function *ε*(*ω*) = *ε*_1_(*ω*) + *iε*_2_(*ω*), where the imaginary part *ε*_2_(*ω*) represents the energy lost through electronic absorption, and the real part *ε*_1_(*ω*) is related to the dielectric constant and describes the electronic-polarizability of the material.^76^ The polarized optical constants along the [100] direction of the plane have been estimated with Gaussian smearing of 0.5 eV, and Drude damping of 0.05 eV. The observed real and imaginary components of the optical dielectric constant are displayed in the uppermost layers of Fig. 7 and 8, respectively.

For BNNS, CO_2_ + BNNS, H_2_S + BNNS, and SO_3_ + BNNS, the real component of the dielectric tensors at zero photon energy *ε*_1_(0) was determined to be 1.676, 1.712, 1.693, and 1.714, respectively. Regarding As-BNNS, *ε*_1_(0) has been determined to be 1.778, 1725, 1.697, and 1.714 for As-BNNS, CO_2_ + As-BNNS, H_2_S + As-BNNS, and SO_3_ + As-BNNS. It shows that during gas adsorption, *ε*_1_(0) of BNNS and As-BNNS revealed moderate alterations. As-BNNS and BNNS nearly always retain a similar route with distinct values as a function of energy in their depicted lines of *ε*_1_(*ω*). For both BNNS and As-BNNS, the highest value of *ε*_1_(*ω*) was determined to be 2.43 and 2.18 respectively. Following gas adsorption, we discovered that the *ε*_1_(*ω*) values for SO_3_ + BNNS, H_2_S + BNNS, and CO_2_ + BNNS are, respectively, 2.47, 2.43, and 2.42. We also determined *ε*_1_(*ω*) to be 2.46, 2.40, and 2.36 for CO_2_ + As-BNNS, H_2_S + As-BNNS, and SO_3_ + As-BNNS, respectively. The sensitive nature of gas molecules is implied by this shift in the maximum values of *ε*_1_(*ω*) caused by gas adsorption. The doping of As in the pure BNNS layer improves the gas sensitivity resulting from the greater chance in *ε*_1_(*ω*) in As-BNNS. The dielectric tensor *ε*_2_(*ω*) in BNNS shows peaks with maxima at around 3.78 eV and stays at zero from 0.0 eV to 1.91 eV when it starts to rapidly rise. This might be explained by charge carriers' intraband transition. Up to 7.5 eV, there is very little movement in the *ε*_2_(*ω*) for the gas-adsorbed BNNS. The gas adsorption in As-BNNS has been reported to cause a significant change of *ε*_2_(*ω*) of about 1.9 eV. For BNNS, CO_2_ + BNNS, H_2_S + BNNS, and SO_3_ + BNNS, the highest *ε*_2_(*ω*) values are 1.50, 1.51, 1.48, and 1.45 respectively. As-BNNS, CO_2_ + As-BNNS, SO_3_ + As-BNNS, and H_2_S + As-BNNS are calculated to be 1.37, 1.47, 1.38, and 1.38 in As-BNNS, respectively. These results are a prime indicator of a shift in *ε*_2_(*ω*) due to gas adsorption by BNNS and As-BNNS.

#### 3.4.2. Refractive index

The spectra of the real component of the refractive index, *n*(*ω*), in both the gas adsorbed and host layers closely resemble the *ε*_1_(*ω*) tensor seen in Fig. 7's middle layer for both BNNS and As-BNNS. The highest values of *n*(*ω*) exhibit variation throughout the energy range of 1.9 eV to 4.33 eV, with a decline beginning at 12.0 eV after two-fold peaks. The square root value of the dielectric function *ε*_1_(*ω*) is comparable to the term “refractive index *n*(*ω*)”, as we saw, and as a result, the graphs for these two parameters resembled one another. As shown in the top and middle layers of Fig. 8, the imaginary component of the refractive index, *k*(*ω*), and incident photon attenuation consisted of *ε*_2_(*ω*). The number of occupied states and photon attenuation are closely correlated. As a result of this process, the number of occupied states increases, increasing the dielectric constant and the attenuation of light photons. A means of identifying the deposited gas might be the fluctuation in refractive index, which indicates that the speed of the photon in the medium varies after gas adsorption.

#### 3.4.3. Absorption coefficient and optical conductivity

To ascertain a 2D layer's sensitivity to gas molecules, the absorption coefficient and optical conductivity analysis may be the most crucial and comprehensible components. The electrical conductivity of a material resulting from electromagnetic radiation is known as optical conductivity, and the percentage of light intensity attenuation per unit of distance that light travels through a medium is known as the absorption coefficient.

The optical conductivity is represented by the real component *σ*_1_, while the absorption loss is represented by the imaginary part *σ*_2_, which is related to *ε*_2_(*ω*). We may achieve a very similar pattern of optical conductivity *σ*_1_(*ω*) and absorption coefficient *α*(*ω*) as seen in Fig. 7 and 9. The first peaks of *σ*_1_(*ω*) and *σ*_2_(*ω*) were made up of the findings from the EBS investigation, and the nonzero values of *α*(*ω*) and *σ*_1_(*ω*) for a certain energy range indicate the presence of a bandgap as shown in EBS analysis. The As doping and gas adsorption cause a change in the beginning point of *σ*_1_(*ω*) and *α*(*ω*). These changes are more pronounced in As-BNNS than in pure BNNS and are attributed to gas adsorption. They are comparable to how bandgap analysis has changed. Remarkably, neither conduction nor absorption took place at lower energies as they are not strong enough to excite electrons; instead, they help molecules vibrate. Both *σ*_1_(*ω*) and *α*(*ω*) show a maximal number of electron transitions at the higher energy range, around 11.4 eV to 17.5 eV, as a result of the wider and bigger spectra. For BNNNS/As-BNNS and gas-adsorbed BNNS/As-BNNS, the highest values of *σ*_1_(*ω*) and *α*(*ω*) are obtained at 12.1 eV and 12.5 eV, respectively. It is evident from the spectra of *σ*_1_(*ω*) and *α*(*ω*) that the gas adsorption causes a rise in their maximum values. A slight peak shift in the absorption spectra is observed due to gas adsorption which can be utilized to identify the type of associated toxic gas.^78^

#### 3.4.4. Reflectivity

The adsorbed gas type may be identified by examining the variance in the reflected energy spectra.^78^ The middle layer of Fig. 9 shows the reflectivity spectra of virgin and gas-adsorbed structures. As is well known, some photons are absorbed, some are transmitted, and others are reflected when they collide with a substance. Since absorption is weaker in the low-energy zone, all structures had greater reflectivity values. As-BNNS has a maximum loss of 6.6% of photon energy at 4.6 eV, whereas BNNS shows a maximum reflectivity of 7.48% of incident photon energy at 4.77 eV. Primarily the image illustrates that the maximum reflectance shifts as a result of gas adsorption. Because of the strong absorption in the UV zone, reflectance tends to zero there. Low-energy reflectivity spectra show a redshift following gas adsorption in BNNS, whereas high-energy region reflectivity spectra show a blueshift. But gas adsorption in the case of As-BNNS results in a blue shift in the reflectivity spectrum at all energies. The shifting in reflection peaks may result in a slight change in the color of the adsorbent due to gas adsorption which can be calibrated to identify the type of toxic gas present in a chamber.^62^

#### 3.4.5. Loss function

One of the most significant peaks in the energy loss function spectrum is the plasmon peak. Connected to this peak are the reflectivity peaks and the imaginary component of the dielectric function, *ε*_2_(*ω*). The highest peaks are referred to as plasmon peaks and the matching frequency is referred to as the plasma frequency because of their collective oscillations. The material becomes transparent to light if the incident light's frequency is higher than the plasma frequency. We noticed a slight blue shift in the plasmon peaks from the bottom layers of Fig. 9 as a result of gas adsorption. Following the dielectric function *ε*_2_(*ω*) the loss peaks were found to be high within the 11.4 eV to 17.5 eV range.

## 4. Conclusions

A tetragonal BN layer has been designed and doped with an As atom to explore their adsorption properties using DFT calculations. Toxic gas molecules such as CO_2_, H_2_S, and SO_3_ are adsorbed successfully with a significant increase in adsorption energy *via* As-doping. Through geometry optimization and the determination that the cohesive energy is negative, the energetically stable configuration of the doped and undoped BNNS layers has been achieved. Greater variations in the bond lengths of the adsorbent and adsorbate suggest a comparatively strong interaction between As-BNNS and selected gases. Compared to CO_2_ and SO_3_, the adsorbent layers interact more with SO_3_, and As-doping enhances this BNNS interaction significantly. As evidenced by the conductivity research, DOS, and EBS results, pristine BNNS is naturally insulating. Gas adsorption reduces the band gap and promotes conductivity. During SO_3_ adsorption, an almost insulator-to-semiconductor transition takes place. In contrast, gas adsorption broadens the bandgap and As-doping turns BNNS into a semiconductor. The adsorbent materials' sensitivity to adsorbates was reinforced by the change in conductivity brought about by the adsorption of gas molecules. The BNNS and As-BNNS exhibit low refractive index and strong UV absorption coefficients, indicating their promise as optoelectronic research materials. These adsorbent layers can function as gas sensors, as demonstrated by the shift in peak positions of reflectivity and absorption throughout the gas adsorption process. A notable alteration in the reflection spectra implies that color changes may be used to identify the adsorbed gases. A thorough examination of the geometrical and optoelectronic characteristics reveals that As-BNNS outperforms BNNS as a gas sensor.

## Data availability

The research data is not shared since it is currently being used in an ongoing study.

## Conflicts of interest

The authors state that none of the work presented in this study may have been influenced by any known conflicting financial interests or personal ties.

## References

[cit1] Wang C., Cui X., Liu J., Zhou X., Cheng X., Peng S., Hu X., Li X., Zheng J., Lu G. (2016). Design of superior ethanol gas sensor based on Al-doped NiO nanorod-flowers. ACS Sens..

[cit2] Zhang J., Liu X., Neri G., Pinna N. (2016). Nanostructured materials for room-temperature gas sensors. Adv. Mater..

[cit3] Vergara A., Kurt D. B., Montgomery C. B., Steve S. (2014). Demonstration of fast and accurate discrimination and quantification of chemically similar species utilizing a single cross-selective chemiresistor. Anal. Chem..

[cit4] Luo C., Tong C., Huang L., Xie L., Qin D., Xiao X. (2024). High-efficiency hydrogen detection for Sc decorated biphenylene based gas sensors: Insights from DFT study. Int. J. Hydrogen Energy.

[cit5] Ahmed M. T., Hasan S., Islam S., Ahmed F. (2023). First principles investigations of Cobalt and Manganese doped boron nitride nanosheet for gas sensing application. Appl. Surf. Sci..

[cit6] Abooali A., Safari F. (2020). Adsorption and optical properties of H2S, CH4, NO, and SO2 gas molecules on arsenene: a DFT study. J. Comput. Electron..

[cit7] Xie L., Chen T., Dong X., Liu G., Li H., Yang N., Liu D., Xiao X. (2023). A comparative study of the electronic transport and gas-sensitive properties of Graphene+, T-graphene, Net-graphene, and biphenylene-based two-dimensional devices. ACS Sens..

[cit8] Dong X., Tong C., Liu G., Xie L., Zhou G., Long M. (2022). Multifunctional 2D g-C4N3/MoS2 vdW heterostructure-based nanodevices: spin filtering and gas sensing properties. ACS Sens..

[cit9] Luo H., Xu K., Gong Z., Li N., Zhang K., Wu W. (2021). NH3, PH3, AsH3 adsorption and sensing on rare earth metal doped graphene: DFT insights. Appl. Surf. Sci..

[cit10] Ali M., Khan S., Awwad F., Tit N. (2020). High gas-sensing selectivity of bilaterally edge-doped graphene nano-ribbons towards detecting NO2, O2 and SO3 gas molecules: *Ab-initio* investigation. Appl. Surf. Sci..

[cit11] Osouleddini N., Rastegar S. F. (2019). DFT study of the CO2 and CH4 assisted adsorption on the surface of graphene. J. Electron Spectrosc. Relat. Phenom..

[cit12] Salih E., Ayesh A. I. (2021). Pt-doped armchair graphene nanoribbon as a promising gas sensor for CO and CO2: DFT study. Phys. E.

[cit13] Siddique S. A., Sajid H., Amjad Gilani M., Ahmed E., Arshad M., Mahmood T. (2022). Sensing of SO3, SO2, H2S, NO2 and N2O toxic gases through aza-macrocycle *via* DFT calculations. Comput. Theor. Chem..

[cit14] Azak H., Gorgul R., Tekin B., Yildiz M. (2019). Calculation of conductive polymer-based SO2 and SO3 gas sensor mechanisms by using the DFT method. J. Mol. Model..

[cit15] Molina A., Escobar-Barrios V., Oliva J. (2020). A review on hybrid and flexible CO2 gas sensors. Synth. Met..

[cit16] Nakate U. T., Yu Y.-T., Park S. (2022). Hydrothermal synthesis of ZnO nanoflakes composed of fine nanoparticles for H2S gas sensing application. Ceram. Int..

[cit17] Dong X., Tong C., Zhou G. (2024). Design high performance field-effect, strain/gas sensors of novel 2D penta-like Pd2P2SeX (X= O, S, Te) pin-junction nanodevices: A study of transport properties. J. Alloys Compd..

[cit18] Luo C., Yang N., Dong X., Qin D., Zhou G., Tong C. (2024). Susceptible Detection of Organic Molecules Based on C3B/Graphene and C3N/Graphene van der Waals Heterojunction Gas Sensors. ACS Sens..

[cit19] Luo C., Chen T., Cen K., Xie L., Dong X., Xiao X. (2024). Transition Metal (Co, V, W, Zr) Single-Atom Decorated Biphenylene for Enhancing the Sensing Performance of SF6 Decomposition Molecules. Langmuir.

[cit20] Volanti D. P., Felix A. A., Orlandi M. O., Whitfield G., Yang D.-J., Longo E., Tuller H. L., Varela J. A. (2013). The Role of Hierarchical Morphologies in the Superior Gas Sensing Performance of CuO-Based Chemiresistors. Adv. Funct. Mater..

[cit21] Gilbertson L. M., Busnaina A. A., Isaacs J. A., Zimmerman J. B., Eckelman M. J. (2014). Life Cycle Impacts and Benefits of a Carbon Nanotube-Enabled Chemical Gas Sensor. Environ. Sci. Technol..

[cit22] Ma H.-Y., Li Y.-W., Yang S.-X., Cao F., Gong J., Deng Y.-L. (2010). Effects of Solvent and Doping Acid on the Morphology of Polyaniline Prepared with the Ice-Templating Method. J. Phys. Chem. C.

[cit23] Castro Neto A. H., Guinea F., Peres N. M. R., Novoselov K. S., Geim A. K. (2009). The electronic properties of graphene. Rev. Mod. Phys..

[cit24] Bandyopadhyay A., Paria S., Jana D. (2018). Tetragonal graphene nanodot as carbon monoxide gas sensor and current rectification device. J. Phys. Chem. Solids.

[cit25] Ahmed M. T., Roman A. A., Roy D., Islam S., Ahmed F. (2024). Phosphorus-doped T-graphene nanocapsule toward O3 and SO2 gas sensing: a DFT and QTAIM analysis. Sci. Rep..

[cit26] Singla M., Jaggi N. (2021). Theoretical investigations of hydrogen gas sensing and storage capacity of graphene-based materials: A review. Sens. Actuators, A.

[cit27] Gautam M., Jayatissa A. H. (2011). Gas sensing properties of graphene synthesized by chemical vapor deposition. Mater. Sci. Eng., C.

[cit28] Li Z., Gao F. (2012). Structure, bonding, vibration and ideal strength of primitive-centered tetragonal boron nitride. Phys. Chem. Chem. Phys..

[cit29] Roy S., Xiang Z., Puthirath A. B., Meiyazhagan A., Bhattacharyya S., Rahman M. M., Babu G. (2021). *et al.*, Structure, properties and applications of two-dimensional hexagonal boron nitride. Adv. Mater..

[cit30] Sajjad M., Feng P. (2014). Study the gas sensing properties of boron nitride nanosheets. Mater. Res. Bull..

[cit31] Khan M. I., Akber M. I., Gul M., Iqbal T., Alarfaji S. S., Mahmood A. (2024). Exploring the sensing potential of Fe-decorated h-BN toward harmful gases: a DFT study. RSC Adv..

[cit32] Shtansky D. V., Matveev A. T., Permyakova E. S., Leybo D. V., Konopatsky A. S., Sorokin P. B. (2022). Recent progress in fabrication and application of BN nanostructures and BN-based nanohybrids. Nanomaterials.

[cit33] Oku T. , Narita I., Koi N., Nishiwaki A., Suganuma K., Inoue M. and Hiraga K., *et al.*, Boron nitride nanocage clusters, nanotubes, nanohorns, nanoparticles, and nanocapsules, BCN Nanotubes and Related Nanostructures, 2009, pp. 149–194, 10.1007/978-1-4419-0086-9_6

[cit34] Roy D. (2021). *et al.*
, Understanding the atomistic origin of the magnetic phases in Cobalt-TM (V, Nb, Ta, Zr, Hf, W) pair co-doped boron nitride monolayer and the hydrogenation effect. Phys. E.

[cit35] Ahmed M. T., Islam S., Ahmed F. (2023). Structural, optical, and electronic properties of boron nitride incorporated mobius carbon nanoribbon: a DFT calculation. Phys. Scr..

[cit36] Jia J. F., Wu H. S., Jiao H. (2006). The structure and electronic property of BN nanotube. Phys. B.

[cit37] Peyghan A. A., Soltani A., Pahlevani A. A., Kanani Y., Khajeh S. (2013). A first-principles study of the adsorption behavior of CO on Al- and Ga-doped single-walled BN nanotubes. Appl. Surf. Sci..

[cit38] Goel N., Kumar M. (2021). Recent advances in ultrathin 2D hexagonal boron nitride based gas sensors. J. Mater. Chem. C.

[cit39] Cadore A. R., Mania E., Alencar A. B., Rezende N. P., de Oliveira S., Watanabe K., Taniguchi T., Chacham H., Campos L. C., Lacerda R. G. (2018). Enhancing the response of NH3 graphene-sensors by using devices with different graphene-substrate distances. Sens. Actuators, B.

[cit40] Sajjad M., Morell G., Feng P. (2013). Advance in novel boron nitride nanosheets to nanoelectronic device applications. ACS Appl. Mater. Interfaces.

[cit41] Gui Y., Li T., He X., Ding Z., Yang P. (2019). Pt Cluster Modified h-BN for Gas Sensing and Adsorption of Dissolved Gases in Transformer Oil: A Density Functional Theory Study. Nanomaterials.

[cit42] Soltani A., Raz S. G., Rezaei V. J., Dehno Khalaji A., Savar M. (2012). Ab initio investigation of Al- and Ga-doped single-walled boron nitride nanotubes as ammonia sensor. Appl. Surf. Sci..

[cit43] Nemati-Kande E., Abbasi M., Mohammadi M. D. (2020). DFT studies on the interactions of pristine, Al and Ga-doped boron nitride nanosheets with CH3X (X=F, Cl and Br). J. Mol. Struct..

[cit44] Xia S.-Y., Lu-Qi T., Jiang T., Sun H., Li J. (2021). Rh-doped h-BN monolayer as a high sensitivity SF6 decomposed gases sensor: A DFT study. Appl. Surf. Sci..

[cit45] Moladoust R., Esrafili M. D., Hosseinian A., Alkorta I., Vessally E. (2018). Adsorption sensitivity of pristine and Al- or Si-doped boron nitride nanoflake to COCl2: a DFT study. Mol. Phys..

[cit46] Long Y., Xia S.-Y., Guo L.-Y., Tan Y., Huang Z. (2022). As-doped h-BN monolayer: a high sensitivity and short recovery time SF6 decomposition gas sensor. Sensors.

[cit47] Sakib M. N., Ahmed T., Shafiulla M. A., Afroj F., Piya A. A., Shamim S. U. D. (2024). Understanding the adsorption performance of TG, T-BN, T-AlN, and T-GaN nanosheets toward the thioguanine anticancer drug *via* DFT calculations. AIP Adv..

[cit48] Ribag K., Houmad M., Ahl Laamara R., Benyoussef A., El Kenz A. (2023). Strain enhances the electrical and photocatalytic properties of tetragonal boron nitride. Optik.

[cit49] Segall M. D. (2002). *et al.*, First-principles simulation: ideas, illustrations and the CASTEP code. J. Phys.:Condens. Matter.

[cit50] Head J. D., Zerner M. C. (1985). A Broyden-Fletcher-Goldfarb-Shanno optimization procedure for molecular geometries. Chem. Phys. Lett..

[cit51] Zhou F., Cococcioni M., Marianetti C. A., Morgan D., Ceder G. (2004). First-principles prediction of redox potentials in transition-metal compounds with LDA+U. Phys. Rev. B:Condens. Matter Mater. Phys..

[cit52] Perdew J. P. (1993). *et al.*, Erratum: Atoms, molecules, solids, and surfaces: Applications of the generalized gradient approximation for exchange and correlation. Phys. Rev. B:Condens. Matter Mater. Phys..

[cit53] Froyen S. (1989). Brillouin-zone integration by Fourier quadrature: Special points for superlattice and supercell calculations. Phys. Rev. B:Condens. Matter Mater. Phys..

[cit54] González-González R., Salas-Zepeda M. G., Tlahuice-Flores A. (2019). New two-dimensional carbon nitride allotrope with 1: 1 stoichiometry featuring spine-like structures: a structural and electronic DFT-D study. Phys. Chem. Chem. Phys..

[cit55] Lucarini V. , Saarinen J. J., Peiponen K.-E., and Vartiainen E. M.. Kramers-Kronig Relations in Optical Materials Research, Springer Science & Business Media, vol. 110, 2005

[cit56] Shamim S. U. D. (2022). *et al.*, Understanding Na-ion adsorption in nitrogen doped graphene oxide anode for rechargeable sodium ion batteries. Appl. Surf. Sci..

[cit57] Ahmed M. T., Roy D., Al Roman A., Islam S., Ahmed F. (2023). A First-Principles Investigation of Cr Adsorption on C8 and B4N4 Nanocage in Aqueous Medium. Phys. Chem. Chem. Phys..

[cit58] Ahmed M. T., Islam S., Ahmed F. (2022). Density functional theory study of Mobius boron-carbon-nitride as potential CH4, H2S, NH3, COCl2 and CH3OH gas sensor. R. Soc. Open Sci..

[cit59] Kalwar B. A., Fangzong W., Soomro A. M., Naich M. R., Saeed M. H., Ahmed I. (2022). Highly sensitive work function type room temperature gas sensor based on Ti doped hBN monolayer for sensing CO2, CO, H2S, HF and NO. A DFT study. RSC Adv..

[cit60] Peng X., Liu D., Zhao F., Tang C. (2022). Gas sensing properties of Mg-doped graphene for H2S, SO2, SOF2, and SO2F2 based on DFT. Int. J. Quantum Chem..

[cit61] Khatun R., Ahmed M. T., Islam S., Hossain Md K., Hossain Md A., Ahmed F. (2021). First-principles investigation of hexagonal boron-carbon-nitride (h-BCN) nanosheet (2D) as gas sensor towards toxic gases (CO, H2S, PH3, SO2, and HCN). Int. J. Comput. Mater. Sci. Surf. Eng..

[cit62] Kumar V., Roy D. R. (2019). Single-layer stanane as potential gas sensor for NO2, SO2, CO2 and NH3 under DFT investigation. Phys. E.

[cit63] Ahmed M. T., Roy D., Roman A. A., Islam S., Ahmed F. (2024). Ab Initio Study of the Graphyne-like γ-SiC Nanoflake for Toxic Gas-Sensing Applications. Langmuir.

[cit64] Yang M., Dai J., Wang L., Li Y., Song Y. (2019). First principles study of structural stability against the distribution of Mg and Al atoms and adsorption behaviors of heavy metals of attapulgite. Comput. Mater. Sci..

[cit65] Tonny I. J., Khatun M., Roy D., Abdullah A. R., Mohammad T. A. (2024). A first-principles investigation of BF3 and ClF3 gas sensing on N-defected AlN nanosheets. AIP Adv..

[cit66] Lata S., Singh P. K., Samadder S. R. (2015). Regeneration of adsorbents and recovery of heavy metals: a review. Int. J. Environ. Sci. Technol..

[cit67] Qureashi A., Pandith A. H., Bashir A., Manzoor T., Malik L. A., Sheikh F. A. (2021). Citrate coated magnetite: A complete magneto dielectric, electrochemical and DFT study for detection and removal of heavy metal ions. Surf. Interfaces.

[cit68] Mohan D., Pittman C. U. (2006). Activated carbons and low cost adsorbents for remediation of tri- and hexavalent chromium from water. J. Hazard. Mater..

[cit69] Bayuo J., Abukari M. A., Pelig-Ba K. B. (2020). Desorption of chromium(VI) and lead (II) ions and regeneration of the exhausted adsorbent. Appl. Water Sci..

[cit70] Kushwaha S. K. (2018). Magnetic and Electronic Properties of The Cu-Substituted Weyl Semimetal Candidate ZrCo2Sn. JPhys Energy.

[cit71] Elias C., Valvin P., Pelini T., Summerfield A., Mellor C. J., Cheng T. S., Eaves L., Foxon C. T., Beton P. H., Novikov S. V., Gil B., Cassabois G. (2019). Direct band-gap crossover in epitaxial monolayer boron nitride. Nat. Commun..

[cit72] Mawwa J., Shamim S. U. D., Khanom S., Hossain M. K., Ahmed F. (2021). In-Plane Graphene/Boron Nitride Heterostructures and Their Potential Application as Toxic Gas Sensors. RSC Adv..

[cit73] Muktadir M. G., Alam A., Piya A. A., Shamim S. U. D. (2022). Exploring the Adsorption Ability with Sensitivity and Reactivity of C12- B6N6, C12-Al6N6, and B6N6-Al6N6 Heteronanocages towards the Cisplatin Drug: A DFT, AIM, and COSMO Analysis. RSC Adv..

[cit74] Rezvani M., Astaraki M., Rahmanzadeh A., Ganji M. D. (2021). Theoretical Assessments on the Interaction between Amino Acids and the G-Mg3N2monolayer: Dispersion Corrected DFT and DFT-MD Simulations. Phys. Chem. Chem. Phys..

[cit75] Aasi A., Aghaei S. M., Panchapakesan B. (2021). Outstanding Performance of Transition-Metal-Decorated Single-Layer Graphenelike BC6N Nanosheets for Disease Biomarker Detection in Human Breath. ACS Omega.

[cit76] Mulliken R. S. (1955). Electronic population analysis on LCAO–MO molecular wave functions. II. Overlap populations, bond orders, and covalent bond energies. J. Chem. Phys..

[cit77] Hossain K., Rabu R. A., Khanom M. S., Hossain Md K., Ahmed F. (2023). First-principles calculations to investigate effect of X+ cations variation on structural, mechanical, electronic and optical properties of the XCdCl3 chloroperovskites. Mater. Sci. Eng., B.

[cit78] Gao C., Zhang Y., Yang H., Liu Y., Liu Y., Du J., Ye H., Zhang G. (2019). A DFT study of In doped Tl2O: a superior NO2 gas sensor with selective adsorption and distinct optical response. Appl. Surf. Sci..

